# The role of fivefold symmetry in suppressing crystallization

**DOI:** 10.1038/ncomms13225

**Published:** 2016-10-25

**Authors:** Jade Taffs, C. Patrick Royall

**Affiliations:** 1 H. H. Wills Physics Laboratory, Tyndall Avenue, Bristol BS8 1TL, UK; 2 School of Chemistry, University of Bristol, Cantock's Close, Bristol BS8 1TS, UK; 3 Centre for Nanoscience and Quantum Information, Tyndall Avenue, Bristol BS8 1FD, UK; 4 Department of Chemical Engineering, Kyoto University, Kyoto 615-8510, Japan

## Abstract

Although long assumed to have an important role in the suppression of crystallization and the development of glassformers, the effect of local fivefold symmetry has never been directly tested. Here we consider whether such suppression of crystallization has a kinetic or thermodynamic nature and investigate its mechanism. We introduce a model in which the degree of fivefold symmetry can be tuned by favouring arrangements of particles in pentagonal bipyramids. We thus show that fivefold symmetry has both kinetic and thermodynamic effects on the mechanism of crystallization to a face-centred cubic crystal. Our results suggest that the mechanism of crystallization suppression is related to the surface tension between fluid and crystal. Interestingly, the degree of fivefold symmetry has little effect on crystal growth rate, suggesting that growth may be only weakly coupled to fluid structure in hard sphere like systems. Upon increasing the fivefold symmetry, we find a first-order transition to an alternative icosahedra-rich phase. At intermediate bias strengths we find a one-component glassformer.

## Abstract (web summary)

The suppression of crystallization due to the appearance of structures with fivefold symmetry is widely adopted, but its kinetic and thermodynamic origin remains elusive. Taffs *et al.* show that fivefold symmetry substantially slows down the nucleation rate but not the crystal growth rate as expected.

## Graphical abstract

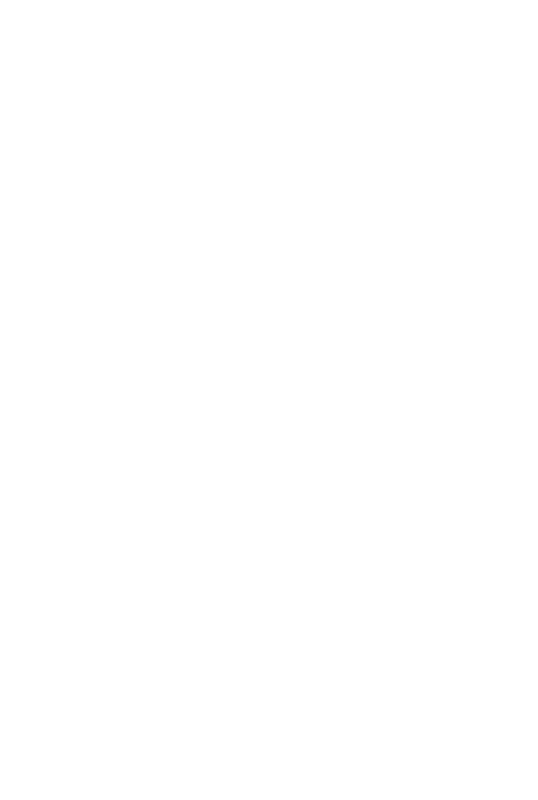

Fivefold symmetry is common in nature, from viruses to atomic liquids, but does not tile Euclidean space^1^,^2^. Over 60 years ago Charles Frank suggested that the formation of energetically favoured, fivefold symmetric icosahedra in supercooled liquids might suppress crystallization, leading to vitrification^1^. Other pioneering studies of liquid structure identified local fivefold symmetry, thus giving rise to the idea that it should have an important role in suppressing crystallization^3^,^4^. This concept of competition between fivefold symmetry in the liquid and sixfold crystalline symmetry underpins the design of materials as diverse as metallic glasses^5^,^6^, quantum liquids^7^, pentagonal molecules^8^ and colloidal polymers^9^.

Despite this longstanding connection between fivefold symmetry and its role in suppressing crystallization, the relationship between liquid structure and crystallization is only just beginning to be addressed^10^ and can often be bewildering, as materials with similar liquid and crystal structures crystallize at markedly different rates^11^. Indirect measurements correlate the prevalence of fivefold symmetric structures with a disinclination to crystallize (or the formation of quasicrystals)^6^,^12^, and some two-dimensional (2d) systems with tuneable frustration have been used to study glass formation^13^,^14^, but in 2d the liquid favours a hexagonal structure like the crystal and the mechanism of crystallization is very different^15^,^16^.

Reflecting the prevalence of fivefold symmetry in the liquid, homogeneous freezing may be interpreted as a competition between fivefold symmetric structures and crystal formation^17^,^18^,^19^. However, other mechanisms have also been proposed, emphasizing crystal-like order in the supercooled liquid^20^,^21^, the formation of locally dense regions^22^ and crystal growth around a fivefold symmetric core^23^. In short, while it is known that fivefold symmetry can suppress crystallsation, the mechanism by which this happens has yet to be addressed, and this forms the subject of our paper.

In particular, a crucial question that underlies the mechanism is whether fivefold symmetry has a role in the rate of nucleation, that is, the nucleation kinetics, as well as altering the equilibrium phase boundaries (for example, by changing composition^6^,^12^). One means by which nucleation may be kinetically suppressed is that local fivefold symmetry in the fluid somehow enhances the fluid-solid surface tension and leading to a higher barrier for a nucleus of given size. Here we use a controlled model system based on hard spheres to systematically vary the fluid's local structure. We show that indeed the kinetics of the nucleation of face-centred cubic (FCC) crystallites is strongly effected by fivefold symmetry and provide evidence that this is related to surface tension.

Our approach varies from that taken to date, which has largely been to observe a system undergoing nucleation and seek evidence in support of a certain mechanism. Here instead of observing a system, we perturb it. This means we can concretely state what the effect of a certain parameter is upon crystallization. To explicitly probe the role of fivefold symmetry in suppressing crystallization, we introduce a model system of hard spheres of diameter *σ* with a many-body biasing potential that can encourage or suppress the formation of pentagonal bipyramids, a basic unit of fivefold symmetry from which larger motifs such as icosahedra^2^ and 10-membered defective icosahedra are comprised^19^. We find that the effect of fivefold symmetry on crystallization has two aspects: thermodynamic and kinetic. First, the fluid-solid phase boundary of the system moves to higher pressure and density under increased fivefold symmetry. Second, for a given degree of supersaturation relative to the phase boundary, the nucleation rate is strongly reduced by an increase in fivefold symmetry, and enhanced when fivefold symmetry is suppressed. Evidence that surface tension is important comes from the shape of crystal nuclei upon varying fivefold symmetry. This rationalizes the empirical observation in a range of experimental systems^6^,^12^,^17^,^18^,^19^, that such packing enhances glass-forming ability, even when measured at fixed supersaturation and after controlling for the effects of the lower melting temperature on the liquid relaxation time at coexistence. Surprisingly, given recent results in binary glassformers^11^, crystal growth rates appear only weakly influenced by the degree of fivefold symmetry in the liquid, while being strong functions of supersaturation. Upon strong biasing towards fivefold symmetry, we find evidence for a dodecagonal quasicrystal or approximant. Between these states we find a eutectic where the system behaves as a one-component glassformer.

## Simulations and model system.

We use Monte Carlo simulations in the isothermal-isochoric (NVT) and isothermal-isobaric (NPT) ensembles. Data is presented for the NVT ensemble unless otherwise stated. To approximate Brownian dynamics, we limit moves to small, single particle displacements with a maximum stepsize of 0.04*σ* (ref. 24). Our hard sphere system is biased towards or away from the formation of pentagonal bipyramids, which we identify using the common neighbour analysis^25^. The pentagonal bipyramid ‘1551' structure comprises a bonded ‘spindle' pair of particles thatshare exactly five, and only five, neighbours. These neighbours form a five-membered ring. We identify bonds using a Voronoi method with a maximum bond length set to 1.4*σ* (ref. 26). Our method thus allows for particles to be part of more than one pentagonal bipyramid. In Supplementary Fig. 1, we show the number of pentagonal bipyramids particles reside in for selected configurations.

Biasing towards (or away from) fivefold symmetry is achieved by associating the formation of each pentagonal bipyramid with an energy penalty or reward of *ɛk*_B_*T*. That is, the field strength *ɛ* denotes the energy change associated with the formation of each individual pentagonal bipyramid structure. A negative *ɛ* encourages pentagonal bipyramid formation, a positive *ɛ* suppresses it. Each new trial configuration is then accepted or rejected according to the Metropolis algorithm. Note that the bias here is applied to instantaneous configurations and is thus distinguished from methods that bias over time-averaged quantities^27^,^28^,^29^.

**Figure 1:**
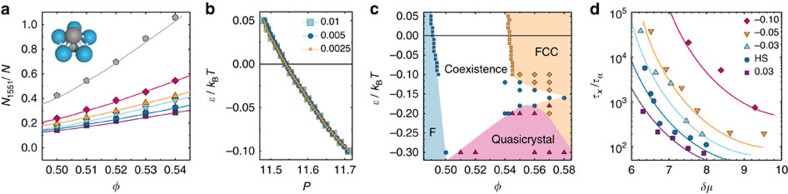
Structure, phase behaviour and crystallization of fivefold symmetric biasing system. (**a**) Number of pentagonal bipyramids (1551) as a function of the volume fraction, *φ*, and bias of the system. (**b**) Coexistence pressure as a function of applied bias, calculated using Gibbs-Duhem thermodynamic integration with *δɛ* as shown. (**c**) Proposed phase diagram. Filled squares are the phase boundaries for fluid-FCC coexistence calculated from Gibbs-Duhem integration. Direct simulation data are shown for fluids (circles), quasicrystal (triangles) and FCC (diamonds). (**d**) Crystallization times, 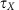, at different strengths of the applied bias are expressed in units of the relaxation time at the phase boundary. Crystallization times are shown as a function of chemical potential with respect to coexistence *δμ* in units of *k*_B_*T*. Lines are to guide the eye. The biasing strength in **a**,**d** is shown in the length in **d**.

Figure 1a shows the number of pentagonal bipyramids as a function of the applied bias. Analysis with the topological cluster classification (TCC) algorithm^26^ shows a correlated change in the higher order structures. The population of clusters rich in five-membered rings, such as icosahedra, also increase as the system is biased towards the formation of pentagonal bipyramids as shown in Supplementary Fig. 2. Conversely, the two-point structure, as shown by the pair correlation function *g*(*r*), is hardly changed by the biasing (see Supplementary Fig. 2a).

## Results

### Disentangling kinetics and thermodynamics

Having demonstrated that we can control the number of pentagonal bipyramids as desired, we need to know two things before we can investigate their effect on crystallization. First, we investigate the effect of biasing on the microscopic dynamics of the particle motion. This is important as it is possible that the system might undergo a change in dynamics upon biasing, and we need to decouple this from the nucleation kinetics. Second, to decouple kinetic and thermodynamic effects on crystallization, we elucidate the position of the phase boundaries between the fluid and FCC crystal phases.

We characterize the dynamics in terms of the intermediate scattering function 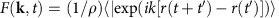 where *ρ* is particle number density and *k* is a wavevector taken close to the first peak in the structure factor. These are fitted with a stretched exponential to obtain the structural relaxation time, 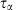. For the values of the bias at which we compare crystallization, −0.1≤*ɛ*≤0.03 *k*_B_*T*, little effect on the dynamics is seen as shown in Supplementary Fig. 3. We scale dynamical data by the relaxation time of the fluid at coexistence 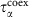 unless otherwise specified.

To investigate the thermodynamic effects of the biasing, we use Gibbs-Duhem thermodynamic integration^30^ to determine phase coexistence in the *P*/*ɛ* plane, where *P* is the pressure. The phase boundary is shown in Fig. 1b. The phase boundary is given by numerical integration along the coexistence curve in steps of *δɛ* (ref. 31), here as a function of *ɛ* starting from hard spheres. Further details are given in the Methods. Figure 1b shows that the coexistence pressure we find is insensitive to the value of *δɛ*. Figure 1c shows a small change in the freezing and melting volume fractions under biasing. Although the absolute change is small, given the sensitivity of the nucleation rate to the volume fraction^32^, accounting for this change is important.

### The role of fivefold symmetry in homogenous crystallization

Having established the effect of the biasing field on the dynamics and phase behaviour, we turn to the nucleation kinetics. We first enquire as to the effect of varying the population of pentagonal bipyramids on homogeneous crystal nucleation. We characterize the nucleation kinetics by defining the crystallization time *t*_*X*_ as the point at which the total crystallinity in a given simulation exceeds, and for the remainder of the simulation stays above, a value of 20%. Crystallinity refers to the proportion of particles identified as FCC or hexagonal close-packed (HCP) with the TCC algorithm.

In the case that the timescales of nucleation and growth are comparable, our definition could lead to confluence of nucleation and growth. However, in our system (as we shall show below), growth is much faster than nucleation. Therefore, our definition effectively tells us the time to form a nucleus. Further details are given in Supplementary Fig. 4. We determined the average crystallization time for each state point, 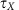, in two ways following^33^. For state points where all simulations crystallized, 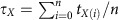 where *n* is the total number of simulations at that state point. On the other hand, for state points where not all runs crystallized, we presume that nucleation is exponentially distributed in time, such that the probability of a nucleation event happening at time *t* is 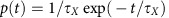. The probability that a given run of length *t*_run_ crystallizes is then 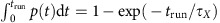. The fraction of runs that crystallized then gives us 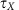.

Here we work in the NVT ensemble, with 2,048 particles, but display our results in Fig. 1d in terms of the change in chemical potential of the metastable fluid with respect to coexistence *δμ*=*μ*−*μ*_coex_ to facilitate comparison with the crystal growth data presented below. The results plotted as a function of the change in volume fraction with respect to freezing are shown in the supplementary information in Supplementary Fig. 5. Noting that the changes in volume fraction and pressure as a function of the biasing field are small (Fig. 1), we find that our system for moderate bias (−0.1≤*ɛ*≤0.03) is well described by the Carnahan-Starling expression for the compressibility factor *Z*(*φ*).

We determine the chemical potential by integrating the equation of state as described in Supplementary Note 1. Figure 1d shows data in line with Frank's hypothesis, that increasing fivefold symmetric structures suppresses crystallization. Moreover, suppressing fivefold symmetry encourages crystallization. By measuring with respect to the phase boundary we can state concretely the effect introducing fivefold symmetry has on the crystallization kinetics.

**Figure 2:**
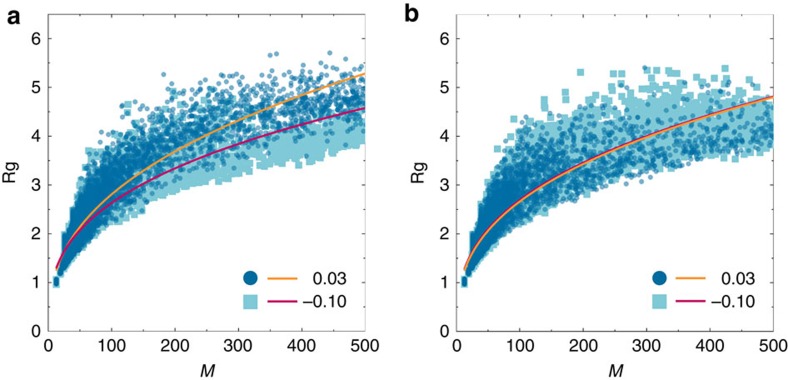
The effect of fivefold symmetry on the shape of crystal nuclei. Comparison of the radius of gyration, *R*_*g*_, of crystal nuclei for systems with an applied bias of 0.03 (circles) and −0.10 *k*_B_T (squares). (**a**) Nuclei are compared at similar supersaturation, *δμ*=8.15 and 8.10 for *ɛ*=0.03 *k*_B_*T* and −0.1 *k*_B_*T*, respectively. (**b**) Nuclei are compared at state points with a similar crystallization time. For *ɛ*=0.03 *k*_B_*T*, *δμ*=6.51 and for *ɛ*=−0.10 *k*_B_*T*, *δμ*=8.99. Data are fitted with a power law, *R*_*g*_=*aM*^*b*^ to obtain the fractal dimension, *d*_*f*_=1/*b*, of the growing crystallites.

We now consider the effect of the biasing field on the nucleation process by examining the geometry of crystalline regions. This enables us to infer the effects of the surface tension between crystal and fluid as fivefold symmetry is varied. Specifically, we determine the radius of gyration, *R*_*g*_, of crystal nuclei, which we plot in Fig. 2 as a function of the number of particles in the nucleus, *M*. To extract the fractal dimension of the nuclei, we fit the data with *R*_*g*_=*aM*^*b*^, where the fractal dimension *d*_*f*_=1/*b*. Given the scatter in the data, and in particular the small size of the nuclei we examine (*M*≤500), our results should be regarded as indicative and comparable with one another rather than accurate measures of *d*_*f*_. We further note that the fractal dimension of a given nucleus may vary considerably from the mean.

Nevertheless for a given supersaturation we expect that increasing fivefold symmetry should increase the interfacial energy between the liquid and crystal because the liquid with enhanced fivefold symmetry has a structure more different to that of the crystal. This favours more compact nuclei. Conversely, in the case that fivefold ordering is suppressed (*ɛ*>0), the interfacial energy should be reduced, and the nuclei more ramified. To test this, we compare the structure of nuclei that form under a bias of either *ɛ*=0.03 or −0.10 *k*_B_*T* under two different conditions. In Fig. 2a, nuclei are compared at similar supersaturation but different field strengths, *δμ*=8.15 and 8.10 for *ɛ*=0.03 and −0.10 *k*_B_*T*, respectively. From fitting, we obtain a fractal dimension of 2.545±0.005 and 2.906±0.013 for *ɛ*=0.03 and −0.10 *k*_B_*T*, respectively. Here the error is the standard error from the fitting of the power law. Thus in line with our expectations, suppression of fivefold symmetry leads to less compact, more ramified nuclei. In Fig. 2b, we consider nucleation at two state points which crystallized over a similar timescale. Given the coupling between crystallization time and field strength, these necessarily occurred at rather different supersaturations, *δμ*=6.51 and 8.99 *k*_B_*T* for *ɛ*=0.03 *k*_B_*T* and −0.10 *k*_B_*T*, respectively. Here the difference in fractal dimension of the crystallites is small, *d*_*f*_=2.727±0.012 for *ɛ*=0.03 *k*_B_*T*, and 2.763±0.010 for *ɛ*=−0.10 *k*_B_*T*. Now the size of the nuclei for *ɛ*=−0.10 *k*_B_*T* is typically smaller, which is reasonable given the system is further from the phase boundary. We thus infer that increasing fivefold symmetry in the fluid leads to an increase in surface tension between fluid and crystal, which acts to suppress nucleation. To fully disentangle the effects of nucleus size and fractal dimension, requires a detailed analysis with larger systems that we leave for the future.

### Crystal growth

We further examine the effects of the biasing field on crystallization kinetics and consider growth. This allows us to investigate the effect of fivefold symmetry at a lower supersaturation than is accessible to our nucleation simulations, and also to investigate whether fivefold symmetry can further suppress crystallization after stable nuclei have formed. Tang and Harrowell^11^ found strong differences in the crystal growth rates of models of NiAl and CuZr even though the models are similar, with identical composition and crystal structure and similar size ratios. Thus, one might imagine that the growth rate may vary significantly due to the structural change in the liquid upon application of the field (Fig. 1). We consider growth on the (111) hexagonal face of the FCC lattice. Compared with either of the (100) or (110) faces, the (111) face has a lower interfacial energy^34^, and more facile growth is expected on this surface^35^. Simulations are carried out in the NPT ensemble to both minimize the build up of stresses and strains within the crystal^36^, and avoid the formation of a depletion zone within the region of the crystal growth front, as may happen in the NVT ensemble^37^. To set-up the growth simulations, we establish a central slab of crystal in contact with the melt either side^11^. We use a cuboid simulation box, of similar dimensions in the *x* and *y* axes, but much longer along the *z* axis. The crystal is arranged such that the (111) faces on which growth will occur are perpendicular to the *z* axis. Details are given in the Methods.

**Figure 3:**
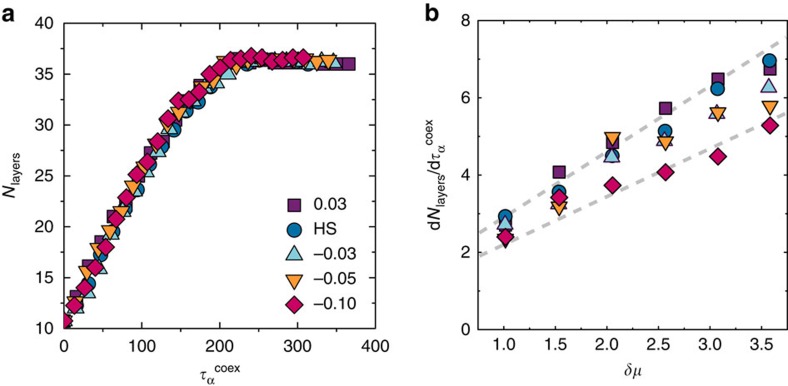
The effect of fivefold symmetry on crystal growth. (**a**) Crystal growth with respect to time for *δμ*≈2.56 *k*_*B*_*T*. For *ɛ*=0.03, *δμ*=2.57 *k*_*B*_*T*, for *ɛ*=0.0, 0.03 *k*_*B*_*T*, *δμ*=2.56 and *ɛ*=−0.05, −0.1 *δμ*=2.55 *k*_*B*_*T*. (**b**) Growth rate with respect to *δμ* for a range of field strength *ɛ*. The dashed lines in **b** are to guide the eye.

To compare growth rates, we measure the number of crystal layers as a function of time (relative to relaxation time at coexistence 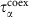). We do not require that the new layer be complete to be counted but set a threshold of a minimum of 10 particles out of a total of 48 (see Methods). To account for the change in phase boundaries with the applied field strength, crystal growth is compared as a function of the change in chemical potential relative to coexistence *δμ*. Results for *δμ*≈2.56 are shown in Fig. 3a. Despite the considerable structural change in the fluid with the changing field strength, we see relatively little change in growth rate at a given degree of supersaturation. As the pressure is increased, Fig. 3b, we see a strong increase in the growth rate with a slight trend to faster growth for reduced fivefold symmetry. This suggests that growth rate and nucleation are decoupled in the sense that the former is little effected by the field, but the field acts to suppress the latter considerably. Therefore, the kinetic effect of fivefold symmetry on crystallization is to suppress homogenous nucleation, not crystal growth.

### Strong bias suppresses FCC ordering

**Figure 4:**
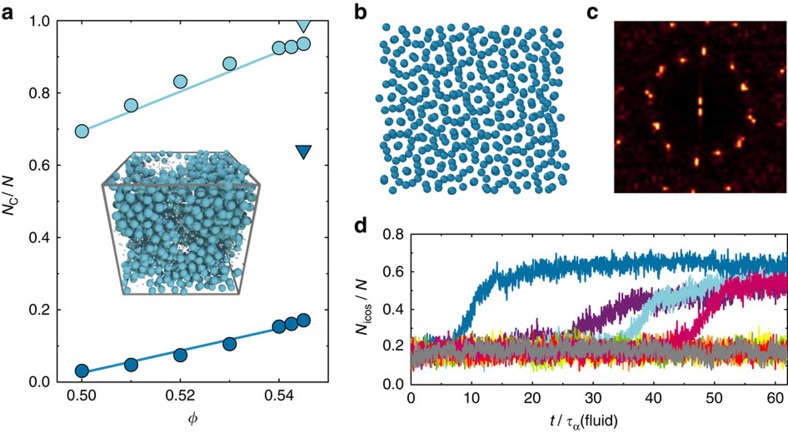
Nucleation of an icosahedra-rich phase. (**a**) Fraction of particles that belong to a pentagonal bipyramid (light blue) or icosahedral structure (dark blue), for systems with a bias of *ɛ*=−0.20 *k*_*B*_*T*. At *φ*=0.545, the system undergoes a transition to an icosahedra-rich state, which is represented by the triangular points. Inset: snapshot of the *φ*=0.545 icosahedra-rich state. Large blue particles are those, which are members of an icosahedron, small grey particles those which are not. (**b**) Plane of the icosahedra-rich state (**c**) A 2d FFT of the projection of the density profile of a configuration of a plane of the icosahedra-rich state shown in **b**. (**d**) Number of particles identified by the TCC as belonging to an icosahedron as a function of time, *φ*=0.545, *ɛ*=−0.2, *N*=2,048.

We now consider the case where the bias is strong, *ɛ*≥−0.10 *k*_B_*T*. One might expect that such a field would suppress crystallization and lead to a one-component glassformer. However, for sufficient volume fraction *φ*≥0.545 and bias 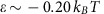 we find two forms of behaviour. Some simulations behaved in a similar way to those at lower supersaturation that is, that they remain in the supercooled liquid state. As shown in Fig. 4a, others formed a state rich in icosahedra. Now this state formed only in some simulations, and the population of icosahedra as a function of time (see Fig. 4d) showed a jump, which suggests a nucleation process. This is further evidenced by the distribution in time at which the jump occurred.

Given that icosahedra do not tile space, one might imagine a transition to an icosahedra-rich phase such as a Frank-Kasper phase as has been noted previously in a model glassformer^38^. In fact, we find a degree of ordering as shown in Fig. 4b. We see a plane of the icosahedra-rich phase and note that a number of particles are surrounded by 12 others. Such ordering is consistent with a 12-fold-symmetric quasicrystal whose local icosahedral structure arises naturally from the biasing field to produce pentagonal bipyramids. To investigate this, we calculate the diffraction pattern (Fig. 4c), which indeed yields 12 peaks. At the level of our analysis, it is unclear as to the exact nature of the state to which the system transitions, but it may be a dodecagonal quasicrystal (or its approximant). Furthermore, although this may be the stable phase, the presence of an underlying FCC crystal cannot be ruled out. Figure 4d shows that under strong bias, our model may provide a suitable means to investigate the nucleation process in quasicrystals. We note that related behaviour has been observed in fivefold symmetric patchy particles^39^, the Dzugutov model^40^, which is also designed to suppress FCC symmetry^41^ and indeed in hard tetraheda, although in that case the local ordering was not fivefold symmetric^42^. These observations lead us to propose the phase diagram shown in Fig. 1c. This shows that for sufficient field strengths, a considerable region of the phase diagram is taken up by the icosahedra rich state. Note that for *ɛ*<−0.1 *k*_B_*T*, we deduce the topology of the phase phase diagram from direct simulation results, unlike the thermodynamic integration we carried out for *ɛ*≥0.1 *k*_B_*T*.

Between the icosahedra rich state and FCC, we find a eutectic (at *ɛ*≈0.16 *k*_B_*T*) where neither is found. Here as expected the system behaves as a one-component glassformer, complementing other models^43^. At strong bias, the system has a somewhat higher relaxation time at a given volume fraction than hard spheres (see Supplementary Fig. 6). Its dynamics are similar to that of polydisperse hard spheres where crystallization is suppressed^44^.

## Discussion

We have introduced a model hard sphere system designed to probe the effect of local fivefold symmetry upon both crystal nucleation and growth by employing a biasing potential to introduce pentagonal bipyramids. This enables us to investigate the mechanism for suppression of crystallization by fivefold symmetry. We find that the effect of fivefold symmetry upon nucleation is both thermodynamic and kinetic. Fivefold symmetry changes the thermodynamics of the system such that freezing is suppressed and the phase boundary, moves to higher pressures and densities. In addition, relative to the phase boundary, increasing fivefold symmetry suppresses the kinetics of homogenous nucleation of FCC crystals. Nucleation is also enhanced by decreasing fivefold symmetry.

By determining the shape of nuclei that form under conditions of enhanced fivefold symmetry, namely that they are rather more compact, we conclude that fivefold symmetry increases the interfacial energy between the crystal and its liquid. This supports the hypothesis that the mechanism by which fivefold symmetry suppresses nucleation kinetics is the enhanced surface tension between fluid and crystal. Interestingly, for a given degree of supersaturation, crystal growth is only weakly dependent on the degree of fivefold symmetry, despite the increase in surface tension. Comparison with crystal growth of glassformers, where a strong change in growth rate was found^11^, suggests more needs to be done before the effects of crystal growth, crucial in exploiting metallic glasses, are fully understood. Our results indicate that in order to suppress crystallinity in glassformers, one should focus on suppressing nucleation of crystalline regions. An interesting question raised by this work is whether other symmetries (such as fourfold or sevenfold) incommensurate with the crystal order might also suppress nucleation.

At strong bias, we find a state rich in icosahedra whose symmetry is consistent with dodecagonal quasicrystal or quasicrystal approximant, although we emphasize that a more systematic study is required before its nature can be confirmed. Our model thus provides a means by which nucleation of quasicrystals may be investigated. Finally, at intermediate bias, a find a eutectic where no ordering occurs: the system behaves a one-component glassformer.

## Methods

### Initial conditions

Each simulation is started from a different set of initial coordinates, with a different seed for the random number generator. With a few high-density exceptions, starting coordinates are limited to having <5% of the particles identified as belonging to a crystalline cluster. The limit is raised to 6% for *φ*=0.55 with a bias of 0 or *ɛ*=−0.01 *k*_B_*T* per ring, 7% for *φ*=0.56, bias *ɛ* =− 0.1 *k*_B_*T* and 8% for *φ*=0.56, bias *ɛ*=−0.05 *k*_B_*T*.

### Gibbs-Duhem integration

The line of coexistence is evaluated as a function of biasing field *ɛ* by integrating the following expression^30^,^31^.


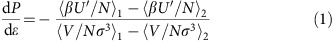


where *U*′ is the partial derivative of the internal energy with respect to the applied bias *ɛ*, and 1 and 2 denote the fluid and (FCC) crystal phases. Simulations are carried out in the NPT ensemble with 256 particles. We take as the starting point a hard sphere coexistence pressure of 11.54 *k*_B_*T*/*σ*^3^, with *φ*_*f*_=0.491 and *φ*_*m*_=0.543 (ref. 36), and a bias *ɛ*=±10^−4^ *k*_B_*T*. The coexistence curve was calculated three times, using stepsizes in *δɛ* of 0.01, 0.005 and 0.0025, the results of which are indistinguishable. The coexistence volume fractions for the two phases at a given *ɛ* are obtained as the ensemble average from NPT simulations of 2,048 particles at the calculated coexistence pressure.

### Crystal growth initial conditions

Starting configurations are prepared from an FCC crystal of 1,728 particles at a volume fraction *φ*=0.548. Particles are arranged in a cuboid box such that the (111) face is parallel to the *xy* plane. The simulation box is then stretched along the *z* axis such that the volume fraction of the system is reduced to *φ*=0.4. An initial, unbiased NVT simulation is run, holding the centre-most FCC layer in place, to rapidly melt the rest of the system. Keeping the central layer held, an NPT hard sphere simulation is run at *P*=12*σ*^3^/*k*_B_*T*, with volume moves limited to changes in the *z* axis only. During this set-up phase, epitaxial nucleation occurs on the pinned crystal surface, and the system begins to contract along the *z* axis. The eight sets of starting coordinates, from which all our simulations are started, are selected from this equilibration simulation. Each is chosen to have between 10 and 11 layers of crystal, which corresponds to just under 1/3 of the length of the final crystal configuration (36 layers of 48 particles). In each case, the *z*—axis lies within a small range of values: 35.304*σ* to 35.314*σ*. For the main simulation, in which the growth is measured, the previously pinned central FCC layer is released, and an NPT simulation is run at the chosen bias strength, and value of *P*_coex_+*δP* where *P*_coex_ is the pressure at coexistence. Volume moves are now allowed in the *xy* plane, as well as along the *z* axis. Eight simulations are run at each state point of interest.

### Data availability

The data from the simulations presented here are available from the corresponding author.

## Additional information

**How to cite this article:** Taffs, J. *et al.* The role of fivefold symmetry in suppressing crystallization. *Nat. Commun.* **7,** 13225 doi: 10.1038/ncomms13225 (2016).

## Supplementary Material

Supplementary Information Supplementary Figures 1-6, Supplementary Note 1 and Supplementary References
