# Supplementary material for: The role of fivefold symmetry in suppressing crystallization
Source: Nat Commun. 2016 Oct 25;7:13225. doi: 10.1038/ncomms13225 (PMC5093329; doi:10.1038/ncomms13225)
Supplement: Supplementary Information — Supplementary Figures 1-6, Supplementary Note 1 and Supplementary References [file ncomms13225-s1.pdf]

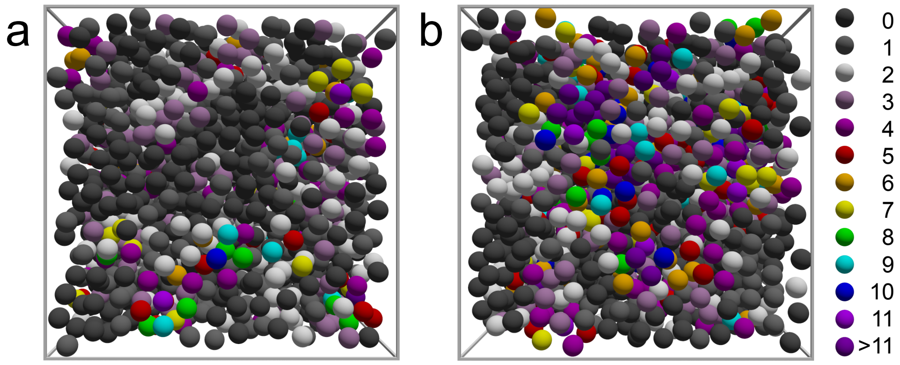

FIG. S1: Snapshots showing the effect of bias on the simulations and that particles can participate in more than one pentagonal bipyramid. Volume fraction $\phi = 0.53$. Particles are rendered 80% of actual size and coloured according to the number of pentagonal bipyramids they participate in as indicated. (a) hard spheres. (b) fluid with enhanced fivefold symmetry: $\varepsilon = -0.1$ k_BT.

I. SUPPLEMENTARY FIGURES

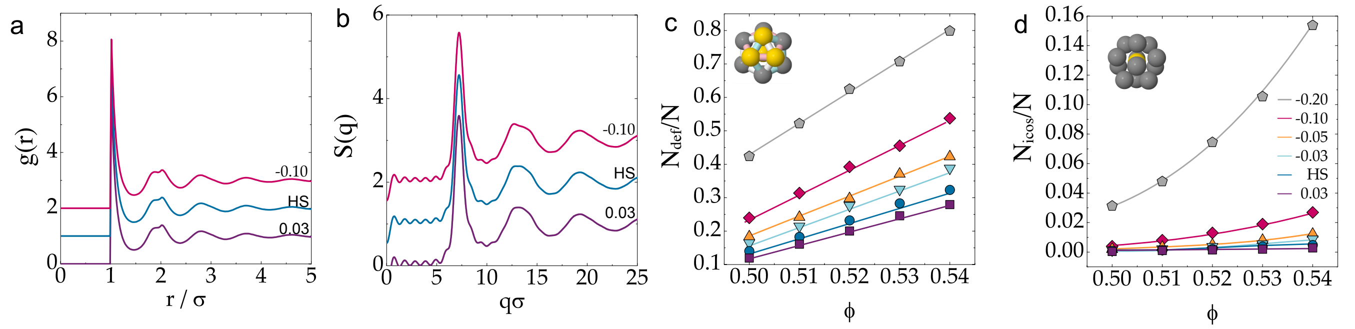

FIG. S2: (a) Radial distribution functions $g(r)$ at various strengths of the biasing field ε as indicated, $\phi = 0.54$. (b) Static structure factor $S(q)$ for the state points in (a). Obtained by Fourier-transforming real space $g(r)$ which leads to weak oscillations for low wavevector q. (c) and (d) Fraction of particles belonging to either a 10-membered defective icosahedron (c) or 13-membered full icosahedron (d) [2] as a function of ϕ for different strengths of the applied biasing field. Legend for (c) is shown in (d).

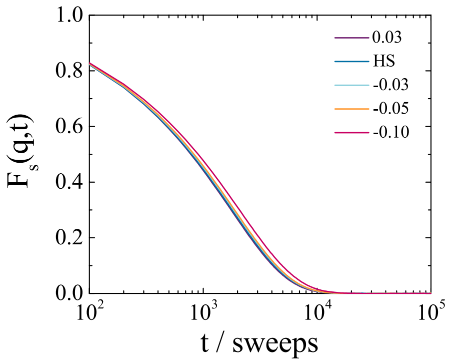

FIG. S3: Intermediate scattering functions $F(k,t)$ for k close to the first peak of the static structure factor. Data are sampled fluids at coexistence for the biasing fields shown. We find rather little effect of the biasing field upon the dynamics.

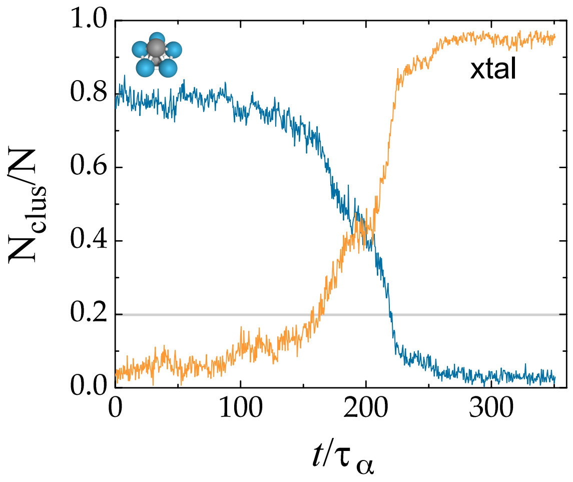

FIG. S4: Structural changes during crystallisation. This run is carried out for $\varepsilon = -0.03$ at a volume fraction $\phi = 0.55$, *i.e.* $\delta\mu = 7.88$. Shown is the population of particles identified in pentagonal bipyraminds and crystalline environments. Grey line denotes the threshold at which we consider the system to be crystallising for the purposes of determining τ_X (20% of the system identified as a crystal).

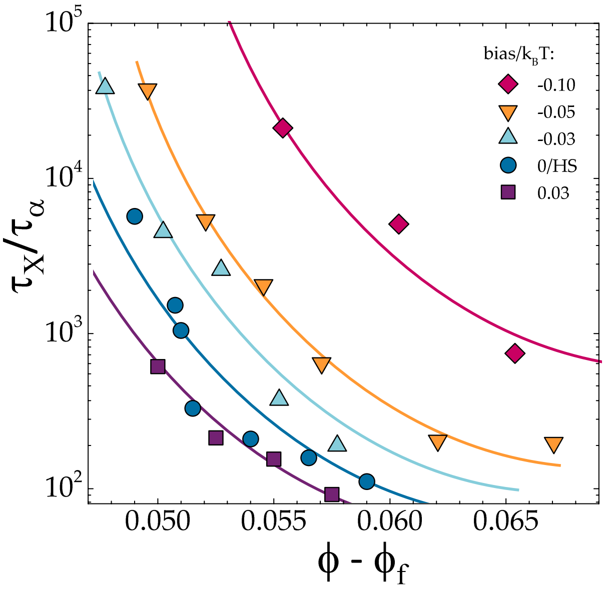

FIG. S5: Crystallisation time τ_X in terms of distance from the melting volume fraction ϕ_m. Data shown corresponds to that in Fig. 1(d) in the main manuscript which is plotted with respect to $p - p_{\mathrm{coex}}$.

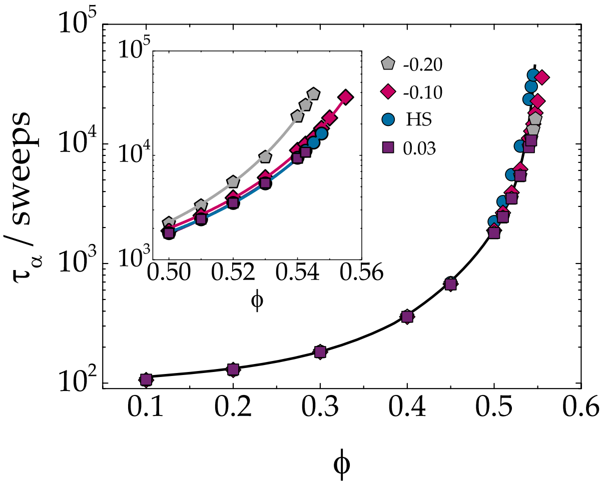

FIG. S6: Structural relaxation time as a function of volume fraction for various biasing strength for various values of the biasing strength. Note that here we plot in Monte Carlo sweeps, rather than the relaxation time at coexistence as elsewhere in this manuscript. At strong bias ($\varepsilon = 0.2$), there is some effect of the bias on the relaxation time compared to weaker biases.

II. SUPPLEMENTARY NOTE 1: DETERMINATION OF CHEMICAL POTENTIAL

We determine the chemical potential relative to the phase boundary $\delta\mu$ by integrating the compressibility factor Z with respect to the volume fraction. This provides the Helmholtz free energy $F(\phi)$.

$$F(\phi,\varepsilon)/V = \frac{6k_BT}{\pi\sigma^3}\phi\int_0^\phi \frac{Z}{\phi'}d\phi' \tag{1}$$

where V is the volume of the system [1]. Adding pressure P then gives the Gibbs free energy $G(\phi,\varepsilon)$ divided by the volume. We take the chemical potential

$$\mu(\phi,\varepsilon) = \frac{G(\phi,\varepsilon)}{N}. \tag{2}$$

To evaluate the change in chemical potential relative to coexistence we assume that for $-0.1 \le \varepsilon \le 0.03$ that the compressibility factor is given by the Carnahan-Starling form. We have performed NPT simulations to determine the equation of state for the biased system and find that within the statistical uncertainties of our data, no deviation from the Carnahan Starling expression is found. We therefore determine the chemical potential difference by integrating Eq. 1 from the phase boundary $\phi^{\rm coex}(\varepsilon)$ for the field strength of interest.

III. SUPPLEMENTARY REFERENCES

[1] W. Russel, D. Saville, and W. Schowalter, *Colloidal Dispersions* (Cambridge Univ. Press, Cambridge,, 1989).
[2] A. Malins, S. R. Williams, J. Eggers, and C. P. Royall, *J. Chem. Phys.* **139**, 234506 (2013).
